# Mass Transfer Analysis of Air-Cooled Membrane Distillation Configuration for Desalination

**DOI:** 10.3390/membranes11040281

**Published:** 2021-04-10

**Authors:** Shuo Cong, Qingxiu Miao, Fei Guo

**Affiliations:** Key Laboratory of Ocean Energy Utilization and Energy Conservation of Ministry of Education, School of Energy and Power Engineering, Dalian University of Technology, No. 2 Linggong Road, Dalian 116024, China; congshuo@mail.dlut.edu.cn (S.C.); miaoqxiu@126.com (Q.M.)

**Keywords:** air-cooled membrane distillation, mass transfer model, characteristic factor, permeate flux

## Abstract

It has been proposed that the air-cooled configuration for air gap membrane distillation is an effective way to simplify the system design and energy source requirement. This offers potential for the practical applications of membrane distillation on an industrial scale. In this work, membrane distillation tests were performed using a typical water-cooled membrane distillation (WCMD) configuration and an air-cooled membrane distillation (ACMD) configuration with various condensing plates and operating conditions. To increase the permeate flux of an ACMD system, the condensing plate in the permeate side should transfer heat to the atmosphere more effectively, such as using a more thermally conductive plate, adding fins, or introducing forced convection air flow. Importantly, a practical mass transfer model was proposed to describe the ACMD performance in terms of permeate flux. This model can be simplified by introducing specific correction values to the mass transfer coefficient of a WCMD process under the same conditions. The two factors relate to the capacity (*B*) and the efficiency (σ), which can be considered as the characteristic factors of a membrane distillation (MD) system. The experimental results are consistent with the theoretical estimations based on this model, which can be used to describe the performance of an MD process.

## 1. Introduction

Membrane distillation (MD) is a membrane-based separation process that only needs low-grade heat as the energy input [1,2,3]. It is a potential desalination technology as the alternative to thermal evaporation technologies [4,5] and membrane filtration technologies [6,7]. MD technology is generally still at the laboratory research stage. The main factors that limit its applications in industrial fields are scale and stability, similar to many emerging technologies. The majority of the studies on MD are focused on the development of novel porous membranes for MD applications [8,9,10,11,12], the transmembrane transport analysis in microscale [13,14,15,16,17], the configurations and their related performance [18,19,20]. The MD performance criteria usually include the permeate flux, salt rejection, specific energy consumption, scaling resistance and operating stability. It is a typical class of research that relates the microscale sciences to engineering applications.

The present work focuses on the MD configuration types that seem to be the most promising for scale-up applications. There are mainly two types of MD systems: gap membrane distillation (GMD) [21,22,23,24,25,26] and direct contact membrane distillation (DCMD) [27,28,29]. Many other MD systems are based on the two configurations [30,31]. They have different advantages but share a common feature that there are two liquid circulations, hot feed and liquid coolant (usually cold water), to provide a temperature gradient as the driving force to make mass transfer across the membrane. The circulation of the hot feed is the necessary part, but the circulation of liquid coolant is a significant drawback of an MD system, which produces an extra energy requirement and system complexity leading to operating costs. The removal of coolant circulation makes the system more compact and cost-effective. Recently, Narayan and Pitchumani [32] creatively proposed an air-cooled membrane distillation (ACMD) configuration, performed experimental investigations on a lab-scale ACMD module, and compared results to those of water-cooled systems in literature [18].

In this work, we investigate the ACMD configuration with a specific focus on the engineering description of an MD process by modeling it with characteristic factors and values. A general mass transfer equation was derived to evaluate and predict MD performance in terms of permeate flux. A series of ACMD modules were constructed, including various condensing plates with different thermal conductivities, finned condensing plates, natural convection and various forced convection configurations. The test results were employed to examine the theoretical estimations and the meanings of the characteristic factors.

## 2. Methods

### 2.1. Membrane Characterization

**Figure 1:**
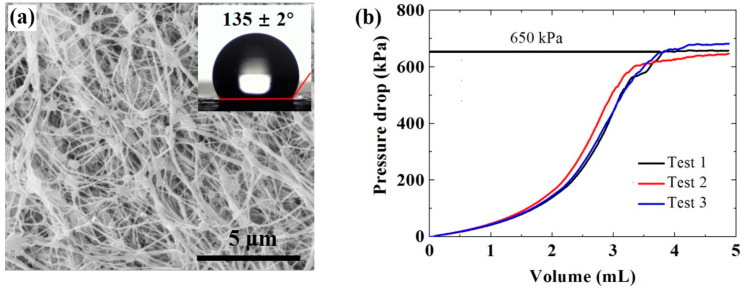
Characterization of the polytetrafluoroethylene (PTFE) membrane used for the membrane distillation (MD) tests. (**a**) The morphology and the wettability in terms of water contact angle. (**b**) Measurement of liquid entry pressure (LEP) value: variation of the pressure drop (kPa) with injected feed liquid volume (mL).

Commercial hydrophobic polytetrafluoroethylene (PTFE) membranes used in this work were purchased from Membrane Solutions LLC. The average pore diameter is 0.22 μm. The porosity is 80%. The surface morphology (Figure 1a) of the hydrophobic membrane was observed by scanning electron microscope (SEM, FEI, Quanta-450, Hillsboro, OR, USA). Due to the low conductivity of the PTFE membrane, the sample was sputter-coated with a thin layer of Au to enhance the conductivity. The thickness of the PTFE membrane was measured by a digital micrometer and averaged. At least six measurements at different locations on the membrane were conducted. The thickness of the PTFE membrane was 153 ± 7 μm.

A goniometer was used to measure the static water contact angle (WCA) of the PTFE membrane to characterize the hydrophobicity. Details of measurements could be found in the previous studies [33,34]. As shown in Figure 1a, the test membrane shows high hydrophobicity (135 ± 2°), which meets the membrane hydrophobicity requirements of MD. Liquid entry pressure (LEP), the minimum pressure to overcome the membrane’s hydrophobicity into the membrane pores, was tested by a custom-designed device. The membrane holder was filled with the membrane. Then a syringe pump was used to generate liquid pressure on the membrane by pumping the DI water slowly (0.5 mL/min). The pressure increased until it exceeded the critical pressure that prevents the liquid from wetting the membrane pores. The detailed LEP tests were described in previous studies [22,35]. As shown in Figure 1b, the LEP value of the PTFE membrane used in this work is ~ 650 kPa, which meets the requirements of MD experiments conducted here.

### 2.2. Membrane Distillation Apparatuses and Tests

**Figure 2:**
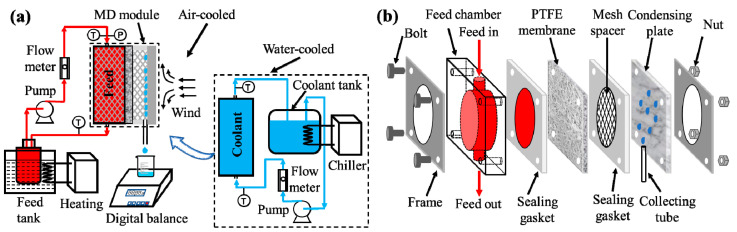
(**a**) Schematic diagrams of the lab-scale air-cooled membrane distillation (ACMD) test unit and water-cooled membrane distillation (WCMD) test unit in this work. (**b**) The expanded diagram of ACMD module configuration.

The ACMD configuration and water-cooled membrane distillation (WCMD) configuration share the same feeding system but differ in terms of cooling system strategy and design (Figure 2). The ACMD module consists of a feed chamber, a test membrane, two sealing gaskets, a mesh spacer, a condensing plate, a collecting tube, two stainless steel frames and four sets of bolt and nut assemblies. The size of the feed chamber 65 × 20 × 65 mm^3^ (length × width × height) made of polymethyl methacrylate (PMMA). It was designed as a circular chamber with a diameter of 50 mm and a depth of 10 mm. The effective membrane area was 17.67 cm^2^. The air gap thickness was 2 mm. The feed side and air gap were both filled with polyethylene mesh spacers for the support of the test membranes. The dimension parameters of the mesh spacer were shown in detail in Table S1. A stainless steel tube was placed between the rubber sheet and the condensing plate to conduct the flow of the permeate water (Figures S1 and S2 of SI). The MD test units in this work applied condensing plates made of different materials, including T2 copper, 1060 aluminum alloy, 304 stainless steel and PMMA. The plates have different thermal conductivities that vary from 0.18–398 W/m/K (Table 1). The thickness of the condensing plates is 1 mm.

In the WCMD configuration (Figure 2a and Figure S1), the hot feed solution was heated by a water bath (DF-101S, Henan Yuhua Instrument Co., Ltd., Henan, China) and pumped into the feed channel by a magnetically driven pump (MP-15R, 10 W, Guangquan Machinery Co., Ltd., Zhengzhou, China). In the cooling side of the WCMD experiment, the cooling water was circulated through the coolant channel to the coolant tank by a magnetically driven pump (30 W) and then cooled to the desired temperature by the chiller.

In the ACMD configuration, the evaporation, transport, and condensation processes of the water molecules were the same as those in WCMD. However, ACMD reduced the use of the pump, chiller, coolant tank, and flow meter on the cooling side. In contrast, natural convection or forced convection of the air by the fan (2.5 W) was employed to transfer the heat from the condensing plate to the atmosphere. The condensing plate was fabricated by flat plates with various thermal conductivities or fins. The permeate water was collected from the lower portion of the MD module, and the change in weight was automatically recorded every 60 s by a digital balance. Besides, two temperature sensors were attached to the outer surface of the condensing plate to measure the temperature of the condensing plate more accurately.

The 3.5 wt% sodium chloride (Tianjin Kemiou Chemical Reagent Co., Ltd., Tianjin, China) aqueous solution was used as the feed. The recirculation flow rate of the feed was maintained at 1.2 L/min. The temperature of the feed was varied in the range of 38 and 71 °C by a water bath. In the WCMD tests, the temperature and the flow rate of the cooling water were maintained at 22.5 ± 0.5 °C and 1.6 L/min, respectively. In the forced convection ACMD tests, the velocities of airflows were set as 0, 0.4, 1.0 and 2.0 m/s, respectively. The relative humidity of the cooling air was ~ 50%. The temperatures of the feed solution and cooling air were maintained at 68.8 ± 0.2 °C and 22.5 ± 0.5 °C, respectively. Each MD test was carried out for 1 h and repeated three times under the same conditions.

To further enhance the driving force of the ACMD configuration, cooling fins were added to the condensing plates, which significantly increased the surface area so that the heat transfer could be increased. Three types of fins were designed (as shown in Figures S3 and S4) and made of aluminum (thermal conductivity: 234 W/m/K). The surface area ratios of fins 1, fins 2, and fins 3 were 14.2, 14.9 and 25.2, respectively. The air velocity was fixed at 2.0 m/s. The specific size parameters of the fins are shown in detail in Table S2.

## 3. Results and Discussion

### 3.1. Feasibility of ACMD Configuration

**Figure 3:**
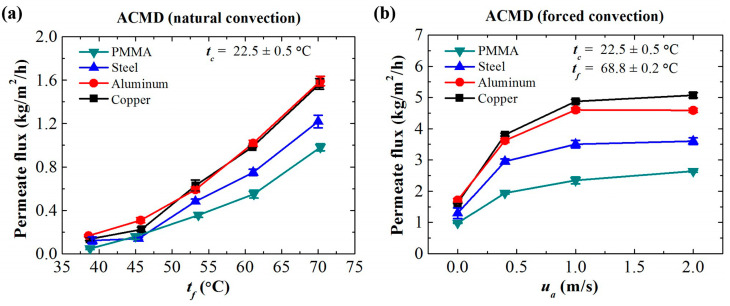
Permeate flux of the ACMD configuration using the condensing plates with various thermal conductivities. (**a**) Natural convection. (**b**) Forced convection: variation of permeate flux with airflow velocity *u_a_* at fixed feed temperature *t_f_* and air temperature *t_c_*.

The ACMD configuration works when there is a temperature gradient between the feed side and the cooling side (Figure 3a). The values of the salt rejection ratio in all the cases in this work are larger than 99% (Table S3). When the temperature of the cooling air is fixed, the permeate flux increases with the temperature of the feed. The trend is similar to that of the WCMD configuration, but the values are small. Besides, the permeate flux of the ACMD process is related to the thermal conductivity of the condensing plate. A plate with higher thermal conductivity leads to a more efficient heat transfer to the atmosphere and a higher permeate flux as a result. The permeate flux increases with the thermal conductivity until a certain point when the natural convection is insufficient to further enhance the heat transfer performance. In this work, the highest permeate flux (1.6 kg/m^2^/h) of the ACMD process is achieved with the feed temperature of 71 °C and using the copper condensing plate under natural convection conditions.

To further increase the permeate flux, forced convection was introduced to the cooling side of the ACMD system to enhance the heat transfer from the condensing plate to the atmosphere. As shown in Figure 3b, the permeate flux can be increased significantly by the forced convection at the cooling side. The convection heat transfer coefficient at the outer surface of the condensing plate increases with the air velocity, whereas the total thermal resistance decreases with the air velocity so that the heat flux and permeate flux are enhanced. The permeate flux reaches the highest value (e.g., Copper plate: 5 kg/m^2^/h) when the velocity of the airflow is 1.0 m/s, which is comparable to that of the WCMD configuration. The continued increase of the air velocity has a slight influence on the permeate flux of the ACMD process. This is because the resistance to convective heat transport becomes lower, and the thermal resistance of the ACMD process is mainly reflected in the air gap. The continued increase of the air velocity has a slight influence on the decrease of the total thermal resistance. The ACMD configuration reaches the maximum cooling capability by air in this case. Besides, the salt rejection ratios under the conditions of different condensing plates and air velocities are both larger than 99.9% (Table S4).

### 3.2. Mass Transfer Model for ACMD Configuration

In the microscale, the mass transport across the porous membrane in an MD process is the diffusion behavior of water molecules through the microchannels forced by the vapor pressure gradient. It depends on pore size, porosity, tortuosity and collision behaviors. In general, all the factors can be summarized as the mass transfer coefficient (*B*) of an MD process, as shown in Equation (1). The *B* value can be considered as a constant for simplification based on the previous study [35], which can also be considered as a characteristic factor of a certain MD process.

(1) $$J=B(P_{f}-P_{c}),$$

where J is the permeate flux of an MD process, B is the general mass transfer coefficient, $P_{f}$ and $P_{c}$ are the saturated vapor pressures of the feed side and cooling side, respectively.

According to the Antoine equation, the saturated vapor pressure is dominated by temperature. The permeate flux of a MD process can be directly expressed by the temperature gradient across the membrane (Equation (2)).

(2) $$J=B\left[\exp(23.1964-\frac{3816.44}{t_{f}+273.15-46.13})-\exp(23.1964-\frac{3816.44}{t_{c}+273.15-46.13})\right],$$

where $t_{f}$ and $t_{c}$ are the temperatures of the feed side and the cooling side, respectively.

In the WCMD configuration, the latent heat of evaporation released by the condensation of the water vapor is transferred to the cooling side through the heat conduction of the condensing plate and convection of the cooling water circulation. The heat transport is mainly affected by the experimental parameters such as the properties of the condensing plate and the circling flow rate of the cooling water ($u_{w}$). As water has a large heat capacity, the thermal resistance of conduction and convection on the cooling side is negligible. The temperature of the membrane surface facing the condensing plate can be considered the same as that of the condensing plate and the cooling water ($t_{c}$). The temperature of the feed ($t_{f}$) is considered as the temperature of the membrane surface directly contacting the feed.

In the ACMD configuration, we started the derivation with the assumption that the feed temperature is equal to the membrane surface temperature, which is the same as that of the WCMD configuration. However, the temperature of the condensing plate is not the same as the temperature of cooling air in the atmosphere because air has a relatively small heat capacity and the heat transfer from solid (condensing plate) to gas (cooling air) is much less efficient than that from solid to liquid (cooling water), although we can apply fins and forced convection to enhance the heat transfer performance. In this case, we introduce a temperature correction coefficient ($\varphi_{c}$) to the temperature of the cooling side ($t_{c}$) to reveal the actual temperature of the condensing plate ($t_{cp}$), which is in the range of the feed temperature and cooling temperature (Equation (3)). Then, the permeate flux of an ACMD process can be rewritten as Equation (4). In a WCMD process, $\varphi_{c}\approx 1$, because of the small thermal resistance in the cooling side.

(3) $$\varphi_{c}=\frac{t_{cp}}{t_{c}}\;(1<\;\varphi_{c}\;<\frac{t_{f}}{t_{c}}),$$

(4) $$J=B\left[\exp(23.1964-\frac{3816.44}{t_{f}+227.02})-\exp(23.1964-\frac{3816.44}{\varphi_{c}t_{c}+227.02})\right],$$

**Figure 4:**
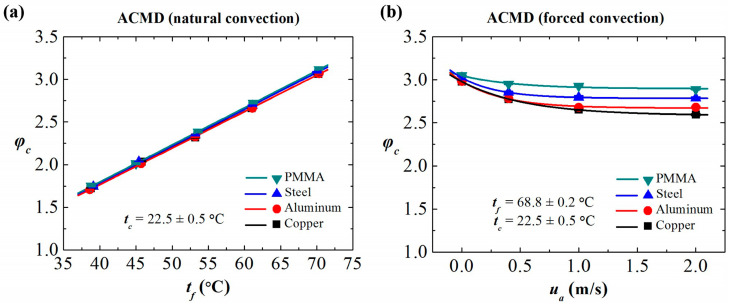
The temperature correction coefficients ($\varphi_{c}$) of the ACMD configuration using the condensing plates with various thermal conductivities. (**a**) Natural convection. (**b**) Forced convection: variation of $\varphi_{c}$ with airflow velocity *u_a_* at fixed feed temperature *t_f_* and air temperature *t_c_*.

As concluded in the previous study [35], the *B* value is a constant for a WCMD process and can be obtained through one set of the MD performance values. Thus, the $\varphi_{c}$ values of an ACMD process can be calculated according to Equation (4). As shown in Figure 4a, in the ACMD processes with various condensing plates, the $\varphi_{c}$ values increase linearly with the feed temperature, $\varphi_{c}\sim t_{f}$. It indicates that natural convection air cooling is not efficient in removing heat from the condensing plate. The temperature gradient across the membrane in an ACMD process is determined by the feed temperature.

As shown in Figure 4b, the forced convection on the cooling side is also a factor influencing the $\varphi_{c}$ value, which is suppressed by the velocity of the airflow ($u_{a}$), following the trend of $\varphi_{c}\sim \exp(1/u_{a})$. This indicates the further cooling on the condensing plate by the forced convection. When the air velocity increases to a certain value (1.0 m/s in this work), the continued increase of the air velocity has an insignificant influence upon the $\varphi_{c}$ of the ACMD process.

It can be concluded that the $\varphi_{c}$ value in Equation (4) varies with the feed temperature and the velocity of the cooling air, which means it is not suitable to be used as a characteristic factor of an ACMD process. Instead, we introduce a specific correction value (σ) to the mass transfer coefficient (*B*) in Equation (2) to generalize the effect of the air cooling to an MD process as follows:

(5) $$J=B\sigma \left[\exp(23.1964-\frac{3816.44}{t_{f}+227.02})-\exp(23.1964-\frac{3816.44}{t_{c}+227.02})\right],$$

In this case, the *B* factor can be considered as a characteristic factor indicating the intrinsic mass transfer coefficient of an MD process. The σ factor can be considered as a characteristic factor indicating the function of air cooling. The value of σ is in the range of 0 to 1. It is approximately equal to 1 in a WCMD process, which is the intrinsic MD performance. In this work, Equation (5) is applied and examined to describe the ACMD performance in terms of permeate flux.

### 3.3. Capacity Factor and Efficiency Factor

**Figure 5:**
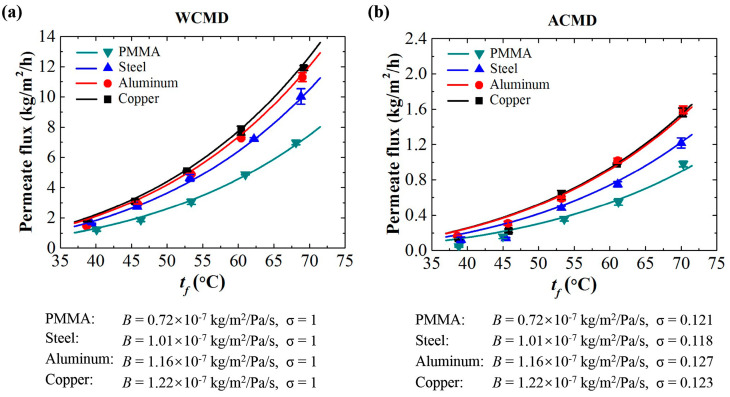
Permeate flux of (**a**) the WCMD configuration and (**b**) the ACMD configuration (natural convection) using the condensing plates with various thermal conductivities. The B value and σ value can be taken as the characteristic factors for an MD system under specific condensing conditions. The solid lines represent the theoretical estimations based on Equation (5). The solid markers represent the experimental results.

As shown in Figure 5, the theoretical estimates of the permeate flux based on Equation (5) are in good agreement with the experimental results of both WCMD configuration and ACMD configuration. For an MD process, regardless of configuration types, the *B* value is fixed as a characteristic factor indicating the intrinsic mass transfer coefficient. The thermal conductivity of the condensing plate is one of the dominant factors affecting the *B* value. Under the same conditions, the condensing plate with higher thermal conductivity results in a larger *B* value which leads to a better MD performance in terms of permeate flux. For an ACMD configuration with the natural convection, the *B* value is the same as that of the related WCMD configuration, while the σ value is about 12% of that. So, the permeate flux of an ACMD test is about 12% to that of a WCMD test under the same conditions. We can conclude that an MD system with fixed intrinsic properties, such as membrane properties and module properties, can be characterized by the *B* value. It represents the capacity of an MD system. The σ value reflects the cooling function of the condensing plate, which represents the efficiency of an MD system. The two factors in Equation (5) can be used to describe and evaluate an MD process of either a WCMD configuration or an ACMD configuration.

### 3.4. Forced Convection ACMD

**Figure 6:**
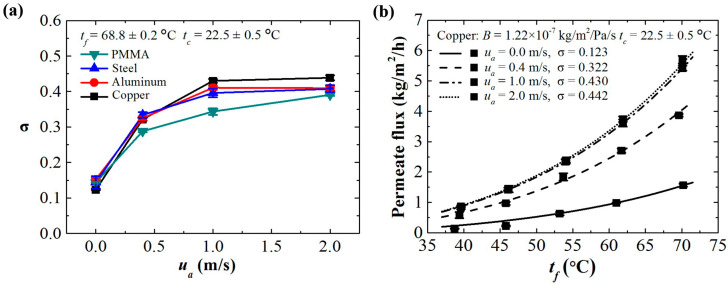
(**a**) The values of the specific correction coefficient of the ACMD configuration using various condensing plates under various air flows for convection heat transfer. (**b**) The theoretical estimations (lines) and the experimental results (solid markers) of the permeate flux of the ACMD system using copper as the condensing plate under air flows.

In the forced convection ACMD configuration, the velocity of the cooling air stream has a significant effect on the efficiency factor (σ). As shown in Figure 6a, the σ value increases with the airstream velocity, which increases the permeate flux. In this work, the σ value can be increased from 0.12 (natural convection) to 0.45 when the velocity of cooling air is 1 m/s by a fan. This is the maximum capacity the system can reach because of the limit of heat transfer. A higher velocity does not increase the σ value in this work. It is clear that forced convection is an effective way to enhance the permeate flux, but the performance is still not as good as that of the water cooling system. We take the ACMD configuration with the copper plate condenser as an example. As shown in Figure 6b, the *B* value is fixed, while the σ values vary with air velocities. The permeate fluxes can be calculated by Equation (5), which are in agreement with experimental results. The model is effective for predicting the permeate flux of MD processes of various forced convection conditions. In this case, the highest permeate flux simply by forced convection air cooling can reach 5.5 kg/m^2^/h.

### 3.5. Effect of Cooling Fins

**Figure 7:**
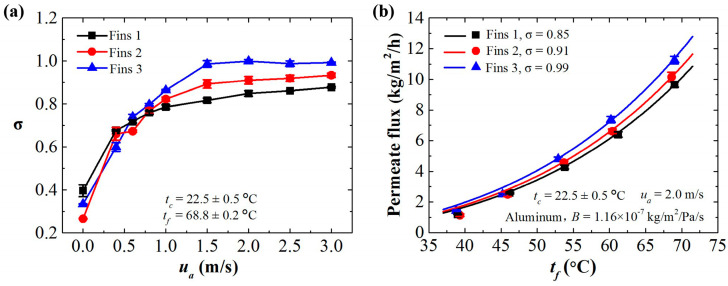
The effect of cooling fins on the permeate flux of the ACMD configuration. (**a**) The specific correction coefficient of the ACMD configuration using an aluminum condensing plate with fins and forced convection. (**b**) The theoretical estimations (solid lines) and the experimental results (solid markers) of the permeate flux of the ACMD system using cooling fins with the optimum airflow velocity (2.0 m/s) in this work.

To further increase the permeate flux of the ACMD configuration, condensing plates with cooling fins can be applied to enhance the heat transfer. As shown in Figure 7a, the finned condensing plate with a higher surface area ratio relates to a larger σ value along with the velocity of the cooling air. The σ value is approximately equal to 1 for the ACMD configuration with fins and forced convection. The efficiency of the ACMD is high, indicating the potential to reveal the intrinsic capacity of an MD system. We take the ACMD system with finned aluminum condensing plate as an example; as shown in Figure 7b, the permeate flux is comparable with that of the WCMD system under the same conditions (Figure 5a). The theoretic estimation according to Equation (5) is consistent with the experimental results. The above results once again verify the effectiveness of the mass transfer model. It provides a convenient way to predict the permeate flux of an MD process, which increases the potential for practical application of MD technology.

## 4. Conclusions

The MD system with the configuration of air cooling can produce permeate fluxes comparable to the liquid cooling configurations by using finned condensing plates and forced convection to improve heat transfer. It is a significant simplification of the original MD configurations with the circulation of cooling water, which leads to the potential practical application of MD using air/wind as the cooling fluid. This is a new route to feature the MD system in more energy-saving and compact structures. We also proposed a new model describing the ACMD performance in terms of permeate flux. We defined the capacity factor (*B*) and the efficiency factor (σ) in the formula. The values of the two factors can be used to characterize an MD system by its intrinsic potential and cooling function, respectively. The theoretical estimation well evaluates the permeate fluxes of MD tests under various conditions. Also, the two characteristic factors well describe an MD system. It is reasonable to believe that the ACMD configuration is a potential route to scaled-up industrial applications.

## Figures and Tables

**Table 1:** The thermal conductivities and water contact angles of the condensing plates used in this work.

	Material	Thermal Conductivity (W/m/K)	WCA (°)
1	Copper	398	46 ± 3
2	Aluminum	234	65 ± 2
3	Steel	18	82 ± 6
4	PMMA	0.18	68 ± 3

## Supplementary Materials

The following are available online at https://www.mdpi.com/article/10.3390/membranes11040281/s1, Figure S1: Photograph of the apparatus used in this work. (a) ACMD experimental apparatus, (b) WCMD experimental apparatus. Figure S2: Photograph of the module used in this work. (a) ACMD module, (b) WCMD module. Figure S3. Schematic diagram of fins structure. $L_{fin}$: length of the fins; $W_{fin}$: width of the fins; $H_{fin}$: height of the fins; $\delta_{ch}$: the fin pitch; $\delta_{fin,1}$: the thickness of the inner fin; $\delta_{fin,2}$: the thickness of the outer fin; $\delta_{base}$: the thickness of the substrate; Ar: surface area of the substrate; Af: side area of the fins. Figure S4. The aluminum finned condensing plates used in this work. Figure S5. Schematic of heat transfer in ACMD process. Table S1. The size parameters of the mesh spacer. Table S2. The size parameters of the fins structure. Table S3. The ACMD performance of the PTFE membrane with various condensing plates at different feed temperatures in natural convection conditions in terms of temperature correction coefficient and salt rejection ratio. Table S4. The ACMD performance of the PTFE membrane with various condensing plates at different air velocities in terms of salt rejection ratio. Table S5. The WCMD performance of the PTFE membrane with various condensing plates at different feed temperatures in terms of salt rejection ratio. Table S6. The ACMD performance of the PTFE membrane with the condensing plate at different surface ratios in the ACMD process.

## Nomenclature

**Symbols**	
Af	side area of the fins (mm^2^)
Ar	surface area of the substrate (mm^2^)
B	mass transfer coefficient of the MD process (kg/m^2^/Pa/s)
$H_{fin}$	height of the fins (mm)
J	permeate flux (kg/m^2^/h)
$L_{fin}$	length of the fins (mm)
N	number of the fin
P	saturated vapor pressure
$R_{s}$	salt rejection ratio
t	temperature (°C)
u	velocity (m/s)
$W_{fin}$	width of the fins (mm)
**Greek letters**	
δ	thickness (m)
$\delta_{base}$	thickness of the substrate (mm)
$\delta_{ch}$	the fin pitch (mm)
$\delta_{fin,1}$	thickness of the inner fin (mm)
$\delta_{fin,2}$	thickness of the outer fin (mm)
λ	thermal conductivity (W/m/K)
σ	specific correction value
φ	temperature correction coefficient
ω	surface area ratio of the condensing plate
**Subscripts**	
*a*	cooling air
*c*	cooling side
*cp*	condensing plate
*f*	feed side
*fin*	rectangular straight fins
*w*	cooling water
**Abbreviations**	
ACMD	air-cooled membrane distillation
GMD	gap membrane distillation
LEP	liquid entry pressure
MD	membrane distillation
PMMA	polymethyl methacrylate
PTFE	polytetrafluoroethylene
RO	reverse osmosis
SEM	scanning electron microscope
WCA	water contact angle
WCMD	water-cooled membrane distillation
