# Supplementary material for: Mass Transfer Analysis of Air-Cooled Membrane Distillation Configuration for Desalination
Source: Membranes (Basel). 2021 Apr 10;11(4):281. doi: 10.3390/membranes11040281 (PMC8069192; doi:10.3390/membranes11040281)
Supplement: Supplementary file 1 [file membranes-11-00281-s001.pdf]

membranes

Supplementary Materials: Mass transfer analysis of air-cooled membrane distillation configuration for desalination

Shuo Cong, Qingxiu Miao and Fei Guo *

School of Energy and Power Engineering, Key Laboratory of Ocean Energy Utilization and Energy Conservation of Ministry of Education, Dalian University of Technology, No. 2 Linggong Road, Dalian 116024, China
* Correspondence: Correspondence: feiguo@dlut.edu.cn

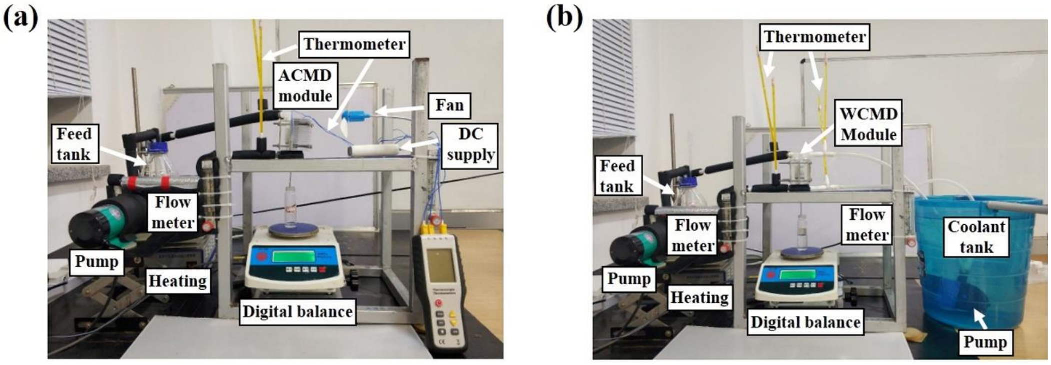

Figure 1. Photograph of the apparatus used in this work. (a) ACMD experimental apparatus, (b) WCMD experimental apparatus.

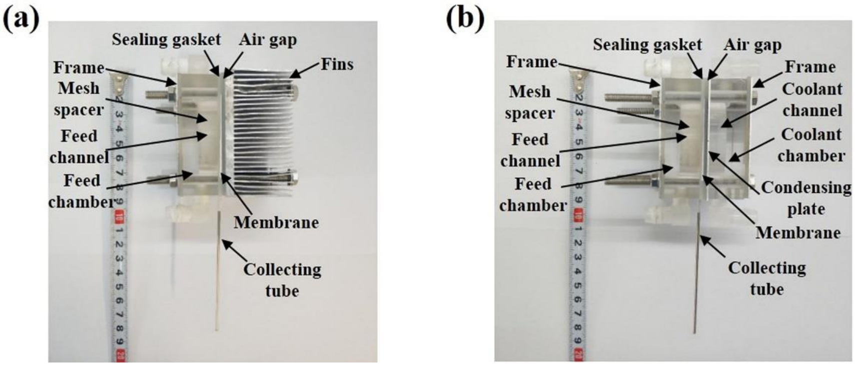

Figure 2. Photograph of the module used in this work. (a) ACMD module, (b) WCMD module.

Table 1. The dimension parameters of the mesh spacer.

Spacer	d_f (mm)	l_m (mm)	h_{sp} (mm)	w_{ch} [a] (mm)	h_{ch} (mm)	θ (°)
	1	4	10	44	10	113

w_{ch} [a] : the average value of the channel width; d_f: filament diameter of the mesh spacer; l_m: the mesh size; h_{sp}: the spacer thickness; h_{ch}: the depth of the channel; θ: the hydrodynamic angle.

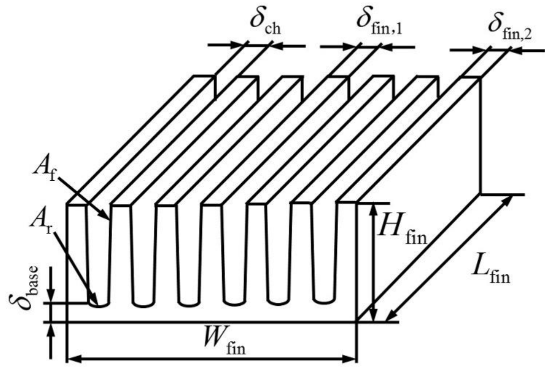

Figure 3. Schematic diagram of fins structure. L_{fin}: length of the fins; W_{fin}: width of the fins; H_{fin}: height of the fins; δ_{ch}: the fin pitch; $\delta_{fin,1}$: the thickness of the inner fin; $\delta_{fin,2}$: the thickness of the outer fin; δ_{base}: the thickness of the substrate; A_r: surface area of the substrate; A_f: side area of the fins.

Table 2. The size parameters of the fins structure.

Fins code	L_{fin}*W_{fin}*H_{fin} (mm)	δ_{base} (mm)	N [b]	$\delta_{fin,1}$ (mm)	$\delta_{fin,2}$ (mm)	δ_{ch} (mm)	A_r (mm^2)	ω [c]
Fins 1	65 × 69 × 45	8.5	13	2.0	2.5	3.75	2795	14.2
Fins 2	65 × 69 × 27	4.6	22	1.2	1.5	2	2730	14.9
Fins 3	65 × 69 × 36	4.6	27	1.0	1.0	1.6	2756	25.2

[b] number of the fin, dimensionless;; ω [c] surface area ratio of the fins, which is the ratio of the total area of the fins (A_r + A_f) to the area of a flat plate with the same size (L_{fin} * W_{fin}), dimensionless.

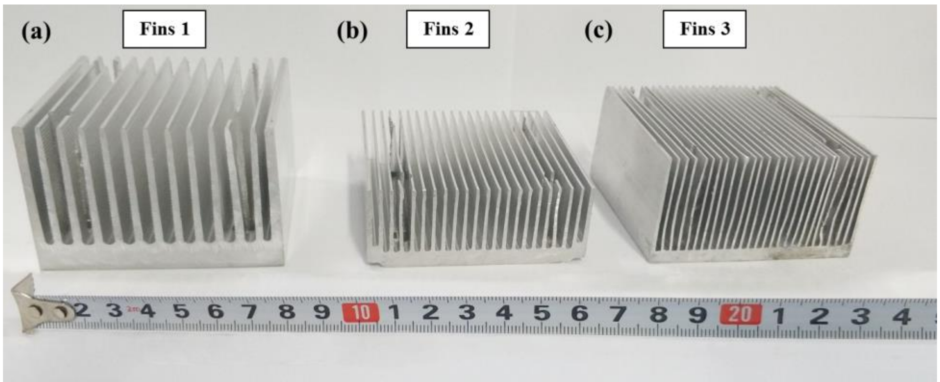

Figure 4. The aluminum finned condensing plates used in this work.

Table 3. The ACMD performance of the PTFE membrane with various condensing plates at different feed temperatures t_f in natural convection condition in terms of temperature correction coefficient φ_c and salt rejection ratio R_s.

Condensing plate materials	t_f (°C)	φ_c	R_s (%)
PMMA	38.8	1.75	99.277
	45.0	2.01	99.451
	53.6	2.38	99.734
	61.2	2.72	99.806
	70.3	3.12	99.811
Steel	39.1	1.74	99.886
	45.4	2.03	99.943

	53.2	2.34	99.960
	61.1	2.69	99.977
	70.0	3.08	99.980
Aluminum	38.6	1.71	99.937
	45.7	2.01	99.889
	53.2	2.33	99.920
	61.1	2.66	99.954
	70.3	3.07	99.957
Copper	38.8	1.72	99.183
	45.8	2.04	99.571
	53.2	2.32	99.634
	61.0	2.66	99.823
	70.2	3.07	99.857

Table 4. The ACMD performance of the PTFE membrane with various condensing plates at different air velocities u_a in terms of salt rejection ratio R_s.

Condensing plate materials	u_a (m/s)	φ_c	R_s (%)
PMMA	0.0	3.06	99.983
	0.4	2.96	99.994
	1.0	2.93	99.994
	2.0	2.88	99.994
Steel	0.0	3.02	99.989
	0.4	2.85	99.997
	1.0	2.80	99.997
	2.0	2.79	99.994
Aluminum	0.0	2.98	99.991
	0.4	2.78	100.00
	1.0	2.68	100.00
	2.0	2.68	100.00
Copper	0.0	2.98	99.991
	0.4	2.78	99.994
	1.0	2.65	99.997
	2.0	2.60	99.997

Table 5. The WCMD performance of the PTFE membrane with various condensing plates at different feed temperatures t_f in terms of salt rejection ratio R_s.

Condensing plate materials	t_f (°C)	R_s (%)
PMMA	40.1	99.651
	46.3	99.600
	53.4	99.714
	61.0	99.766
	68.1	99.791
Steel	39.4	99.880
	45.8	99.983
	53.2	99.940
	62.2	99.986
	68.8	99.946
Aluminum	38.7	99.983
	45.9	99.886

	53.4	99.989
	60.4	99.989
	69.0	99.991
Copper	38.8	99.994
	45.5	99.997
	52.8	99.997
	60.4	99.997
	69.2	99.997

Table 6. The ACMD performance of the PTFE membrane with the condensing plate at different surface area ratios in ACMD process.

Condensing plate	u_a (m/s)	t_{cp} (°C)	R_s (%)
Fins 1 (ω = 14.2)	0.0	59.1	99.989
	0.2	43.9	99.997
	0.4	44.0	99.997
	0.6	43.2	99.997
	0.8	40.5	99.997
	1.0	38.8	99.997
	1.5	36.3	99.997
	2.0	33.8	99.997
	2.5	32.0	99.997
	3.0	30.9	99.994
Fins 2 (ω = 14.9)	0.0	61.5	99.991
	0.2	51.3	99.997
	0.4	46.5	100.00
	0.6	45.5	100.00
	0.8	43.7	100.00
	1.0	40.7	100.00
	1.5	37.3	100.00
	2.0	33.3	99.997
	2.5	33.0	99.997
	3.0	32.0	99.997
Fins 3 (ω = 25.2)	0.0	61.3	99.949
	0.2	50.8	99.963
	0.4	47.8	99.969
	0.6	44.3	99.971
	0.8	42.6	99.974
	1.0	40.4	99.974
	1.5	35.6	99.980
	2.0	31.9	99.989
	2.5	31.5	99.997
	3.0	31.1	99.997

Heat transfer in the MD process

As shown in Fig. S5, heat transfer in the MD process can be divided into the following sections: (1) heat transfer through the hydrophobic membrane (Q_m), including the conduction of the membrane and the latent heat carried by the water vapor; (2) heat transfer through the air gap (Q_{ag}), including the conduction or natural convection of the air and the latent heat of the water vapor; (3) heat transfer by condensation at the inside surface of the condensing plate (Q_{cf}); (4) heat transfer by conduction through the con-

densing plate (Q_{cp}); and (5) heat transfer by convection of the air or water at the outer surface of the condensing plate (Q_c).

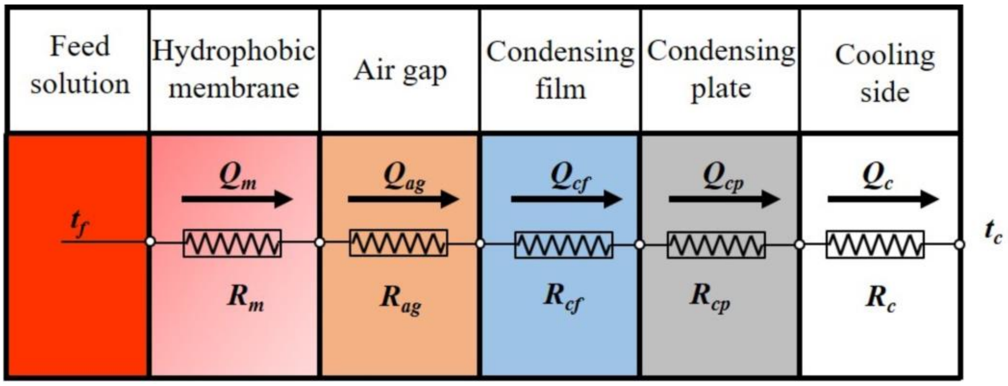

Figure 5. Schematic of heat transfer in ACMD process.

When the MD process was in steady-state, the heat flux through each section should be consistent:

$$Q = Q_m = Q_{ag} = Q_{cf} = Q_{cp} = Q_c = H(t_f - t_c), \quad \text{(S1)}$$

where t_f and t_c are the temperature of the feed side and cooling side, respectively. Q is the heat flux. H is the total heat transfer coefficient in the MD process, which can be calculated by:

$$H = (R_m + R_{ag} + R_{cf} + R_{cp} + R_c)^{-1}, \quad \text{(S2)}$$

where R_m, R_{ag}, R_{cf}, R_{cp}, and R_c represent the thermal resistance through the hydrophobic membrane, air gap, condensing film, condensing plate, and cooling side, respectively.

Next, the investigation was focused on the calculation equation of H under different cooling conditions. When the natural convection of the air is adopted on the cooling side, H is calculated as follows:

$$H = (R_m + R_{ag} + R_{cf} + \frac{\delta_{cp}}{\lambda_{cp}} + \frac{l_{cp}}{0.59\lambda_a (GrPr)^{1/4}\omega})^{-1} = (\mathrm{a} + \frac{\delta_{cp}}{\lambda_{cp}} + \frac{\mathrm{b}}{\lambda_a Gr^{1/4}\omega})^{-1} \quad \text{(S3)}$$

where δ_{cp} and λ_{cp} are the thickness and thermal conductivity of the condensing plate, respectively. λ_a, Gr, and Pr are the thermal conductivity, Grashof number, and Prandtl number of the air, respectively. ω is the effective surface area ratio of the condensing plate. l_{cp} is the characteristic length of the condensing plate. Both a and b keep constant under the given condition.

When the forced convection of the air is applied on the cooling side, H is expressed by:

$$H = (R_m + R_{ag} + R_{cf} + \frac{\delta_{cp}}{\lambda_{cp}} + \frac{l_{cp}}{0.228\lambda_a (\frac{u_a l_c}{v_a})^{0.731} Pr^{1/3}\omega})^{-1} = (\mathrm{a} + \frac{\delta_{cp}}{\lambda_{cp}} + \frac{\mathrm{c}}{\lambda_a u_a^{0.731}\omega})^{-1} \quad \text{(S4)}$$

where u_a and v_a are the velocity and kinematic viscosity of the air, respectively. c is a constant under the given condition.

When the cooling water circulation is employed on the cooling side, H is expressed by:

$$H=(R_m+R_{ag}+R_{cf}+\frac{\delta_{cp}}{\lambda_{cp}}+\frac{l_{cp}}{0.664\lambda_w(\frac{u_w l_c}{\nu_w})^{0.5}Pr^{1/3}})^{-1}=(\mathrm{a}+\frac{\delta_{cp}}{\lambda_{cp}}+\frac{\mathrm{d}}{\lambda_w u_w^{0.5}})^{-1} \quad \text{(S5)}$$

where λ_w, u_w, and ν_w are the thermal conductivity, velocity, and kinematic viscosity of the cooling water, respectively. d is a constant under the given condition.

Besides, when the fins are used as the condensing plate, fin efficiency (η_f) is employed to characterize the effective degree of the fins' heat dissipation. It can be calculated by:

$$\eta_f=\frac{\mathrm{th}\left[\left(2h_c/\lambda_{fin}\delta_{fin}\right)^{0.5}(H_{fin}-\delta_{base}+\delta_{fin}/2)\right]}{\left(2h_c/\lambda_{fin}\delta_{fin}\right)^{0.5}(H_{fin}-\delta_{base}+\delta_{fin}/2)}=\frac{\mathrm{th}\left(h_c^{0.5}/\theta\right)}{\left(h_c^{0.5}/\theta\right)} \quad \text{(S6)}$$

where λ_{fin} is the thermal conductivity of the fins. θ is a constant under the given condition.

In this case, the effective surface area ratio (ω') of the condensing plate is written as follows:

$$\omega'=(\omega-1)\eta_f+1=(\omega-1)\frac{th(h_c^{0.5}/\theta)}{(h_c^{0.5}/\theta)}+1 \quad \text{(S7)}$$

Therefore, the expressions of the total heat transfer coefficient in the MD process under different cooling conditions are summarized as follows:

$$H=\begin{cases}(\mathrm{a}+\frac{\delta_{cp}}{\lambda_{cp}}+\frac{\mathrm{b}}{\lambda_a Gr^{1/4}\omega})^{-1}, & \text{Natural convection of the air}\\ (\mathrm{a}+\frac{\delta_{cp}}{\lambda_{cp}}+\frac{\mathrm{c}}{\lambda_a u_a^{0.731}\omega})^{-1}, & \text{Forced convection of the air}\\ (\mathrm{a}+\frac{\delta_{cp}}{\lambda_{cp}}+\frac{\mathrm{d}}{\lambda_w u_w^{0.5}})^{-1}, & \text{Forced convection of the water}\end{cases} \quad \text{(S8)}$$
